# Exploring the role and mechanism of methionine metabolism in chronic rhinosinusitis with nasal polyps based on bioinformatics analysis

**DOI:** 10.1016/j.bjorl.2026.101917

**Published:** 2026-10-31

**Authors:** Dan Lin

**Affiliations:** The First Affiliated Hospital of Henan University of Chinese Medicine, Department of Otolaryngology, Zhengzhou, China

**Keywords:** Chronic rhinosinusitis with nasal polyps, Methionine metabolism, CSTB, IGFBP3, TCN1

## Abstract

**Objective:**

Chronic Rhinosinusitis with Nasal Polyps (CRSwNP) affects 1%–4% of the global population with high recurrence rates. While methionine metabolism abnormalities contribute to various inflammatory diseases, their role in CRSwNP pathogenesis remains unclear. This study aimed to systematically investigate the role of methionine metabolism in CRSwNP and identify potential biomarkers and therapeutic targets through integrating multi-omics data and interpretable machine learning approaches.

**Methods:**

CRSwNP transcriptomic datasets (GSE136825, GSE179265) and single-cell data (GSE276503) were obtained from GEO database. Single-cell RNA sequencing analysis, differential expression analysis using limma, and Weighted Gene co-expression Network Analysis (WGCNA) were performed to identify methionine metabolism-related candidate genes. Three machine learning algorithms (LASSO, SVM-RFE, XGBoost) with SHAP interpretation screened core genes. Functions and mechanisms were explored through single-gene GSEA, ceRNA network construction, transcription factor prediction, immune infiltration analysis, and pseudotime trajectory analysis. Potential therapeutic drugs were identified via molecular docking.

**Results:**

Among 14 candidate genes, CSTB, IGFBP3, and TCN1 were identified as core genes with excellent diagnostic performance (AUC > 0.85). SHAP analysis revealed CSTB and IGFBP3 as disease risk-promoting factors, while TCN1 showed protective effects. Single-cell analysis demonstrated all three genes were predominantly expressed in epithelial cells with significant differential expression patterns. They were enriched in primary immunodeficiency pathways and significantly correlated with immune cell infiltration, particularly M2 macrophages and activated dendritic cells. Molecular docking identified five potential therapeutic drugs including ambroxol, carbamazepine, and curcumin with favorable binding energies.

**Conclusion:**

Methionine metabolism participates in CRSwNP pathogenesis by regulating CSTB, IGFBP3, and TCN1, affecting epithelial cell function and immune microenvironment. These genes represent promising biomarkers for diagnosis and potential therapeutic targets, providing theoretical basis for developing metabolic-immune intervention strategies in CRSwNP management.

**Level of Evidence:**

3.

## Highlights

• CSTB, IGFBP3, and TCN1 are core methionine metabolism-related genes in CRSwNP.
• These three genes show excellent diagnostic performance with AUC values > 0.85.
• CSTB and IGFBP3 promote disease while TCN1 exhibits protective effects.
• All three genes predominantly expressed in epithelial cells in CRSwNP.
• Methionine metabolism regulates immune microenvironment through M2 macrophages.

## Introduction

Chronic Rhinosinusitis with Nasal Polyps (CRSwNP) affects 1%–4% of the global population, severely impairing quality of life.^1^^,^^2^ Despite therapeutic advances, high recurrence rates persist due to incomplete understanding of pathophysiology.^3^^,^^4^ Recent studies implicate immune dysregulation, epithelial barrier dysfunction, and metabolic reprogramming.^5^^,^^6^

Methionine, an essential amino acid, participates in protein synthesis, methylation modification, cell proliferation, and immune regulation.^7^^,^^8^ The methionine cycle produces S-Adenosylmethionine (SAM), affecting DNA methylation and histone modification.^9^^,^^10^ Abnormal methionine metabolism contributes to inflammatory bowel disease and rheumatoid arthritis,^11^^,^^12^ influencing immune cell activation and inflammatory responses.^13^^,^^14^ However, its role in CRSwNP pathogenesis remains unclear.

Machine learning algorithms (LASSO, SVM-RFE, XGBoost) identify key features from high-dimensional data, while SHAP values enable model interpretation.^15^ Weighted Gene Co-expression Network Analysis (WGCNA) identifies co-regulatory gene modules,^16^^,^^17^ and ceRNA network analysis elucidates gene regulation mechanisms.^18^^,^^19^

Unlike previous bioinformatics studies on CRSwNP that focused on immune dysregulation or EMT pathways, this study is the first to systematically examine methionine metabolism as an organizing framework, identify disease biomarkers, and explore therapeutic targets.

## Methods

### Data acquisition

Bulk RNA-seq datasets GSE136825 (training: 42 CRSwNP, 28 controls) and GSE179265 (validation: 17 CRSwNP, 7 controls), and single-cell dataset GSE276503 (15 CRSwNP, 2 controls) were obtained from GEO. Methionine metabolism genes (n = 1246, relevance score > 7) were retrieved from GeneCards.

### Single-cell analysis

Data were processed using Seurat (v4.0.1) with quality filters (nFeature_RNA < 6000, nCount_RNA < 50000, percent.mt < 2.5%, percent.RP < 0.5%). After normalization and identifying 2000 variable genes, PCA and UMAP dimensionality reduction enabled Louvain clustering. Cell types were annotated using reference databases. Differential expression used FindMarkers. CellChat analyzed cell-cell communication.

### Differential expression and WGCNA

DEGs were identified using limma (|FC| > 2, FDR < 0.05). WGCNA identified disease-associated modules with soft threshold β = 6, minimum module size = 30, cutting height = 0.4. Hub genes required |MM| > 0.6 and |GS| > 0.6. Candidate genes represented intersections of single-cell DEGs, bulk DEGs, WGCNA hub genes, and methionine metabolism genes.

### Functional analysis

GO and KEGG enrichment used DAVID. PPI networks were constructed via STRING and visualized with Cytoscape.

### Machine learning feature selection

XGBoost, LASSO cross-validation, and SVM-RFE identified key genes at their intersection. SHAP values interpreted feature importance. ROC curves evaluated diagnostic performance. Monocle performed pseudotime trajectory analysis.

### Regulatory network construction

ceRNA networks predicted miRNA-mRNA (miRWalk) and lncRNA-miRNA (starBase) interactions. TF networks used miRnet. Networks were visualized with Cytoscape.

### Immune infiltration and clinical model

CIBERSORTx estimated immune cell composition. Wilcoxon tests assessed infiltration differences; Pearson correlation evaluated gene-immune cell relationships. A diagnostic nomogram was constructed using the “rms” package, evaluated by ROC/AUC and decision curve analysis.

### Drug prediction

Candidate drugs were predicted via Enrichr (DSigDB) and CMAP. Molecular docking used AutoDock Vina with structures from PubChem.

## Results

### Identification of methionine metabolism-related candidate genes

Single-cell analysis of GSE276503 identified 19 clusters after quality control and normalization, selecting the first 20 PCs for downstream analysis (Fig. 1A‒C). Based on marker genes (Fig. 1D), six major cell types were annotated: MNPs_DCs, Mast Cells, B_Plasma_Cells, T_and_ILCs, Stromal Cells, and Epithelial Cells (Fig. 1E). Notably, the proportion of epithelial cells was significantly reduced in CRSwNP compared to healthy controls (Fig. 1F). Differential expression analysis using FindMarkers identified 1640 CRSwNP-associated genes (p < 0.05, |avg_log₂FC| > 0.25), which were carried forward into downstream screening.

**Fig. 1:**
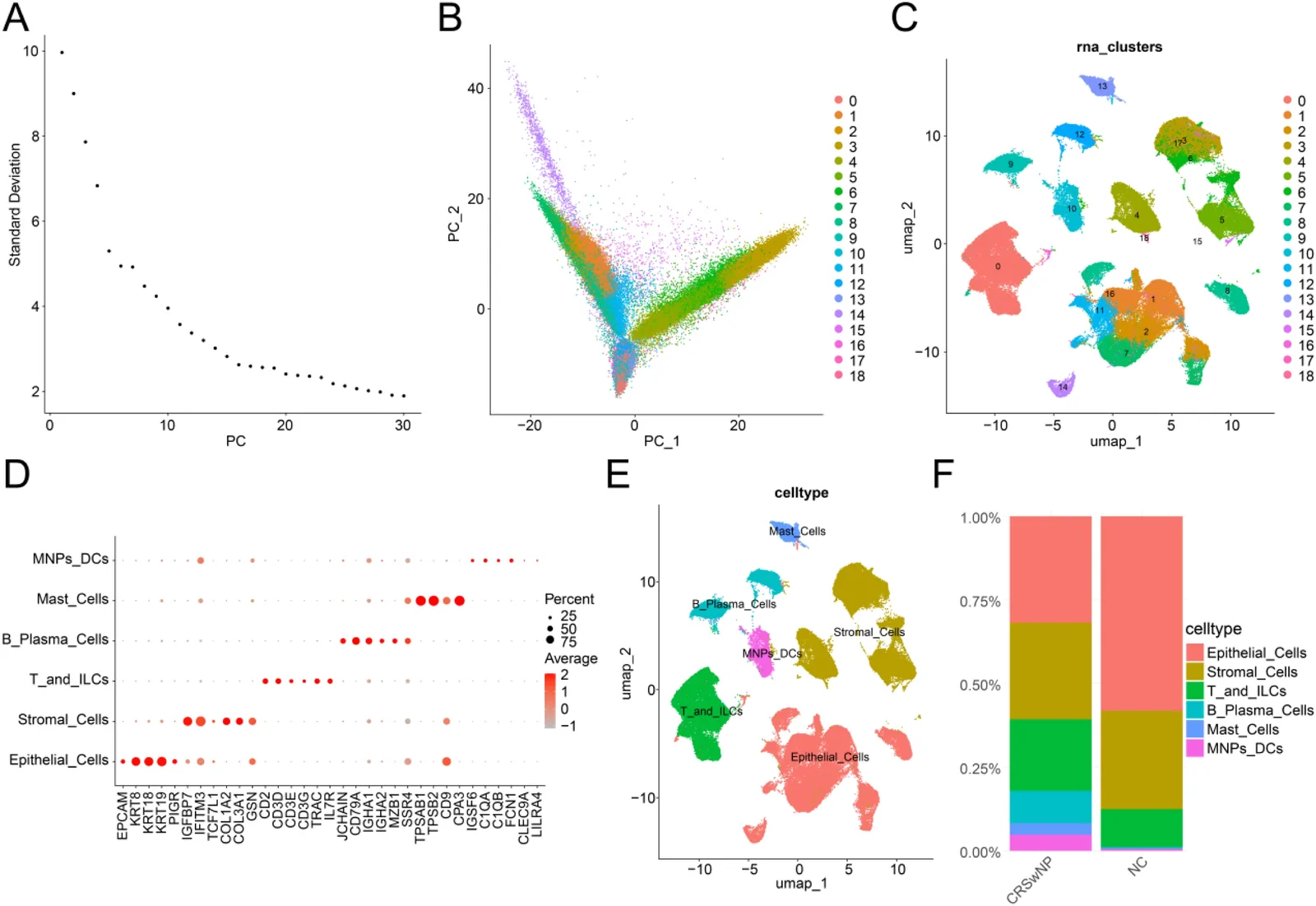
Clustering analysis and cell type annotation of single-cell RNA sequencing data. (A) PCA variance plot; (B) PCA clustering; (C) UMAP clustering; (D) Marker gene heatmap; (E) Cell type annotation; (F) Cell proportion in CRSwNP vs NC.

Bulk RNA-seq differential analysis of GSE136825 identified 2255 DEGs, comprising 1612 upregulated and 643 downregulated genes in CRSwNP (Fig. 2A). WGCNA was subsequently performed with an optimal soft-thresholding power of β = 6 (Fig. 2B), generating 13 co-expression modules (Fig. 2C–D). Nine of these modules showed significant correlation with disease status (Fig. 2E), collectively providing 4663 hub genes (|MM| > 0.6 and |GS| > 0.6). The convergence of four independent analytical approaches (single-cell DEGs, bulk DEGs, WGCNA hub genes, and methionine metabolism gene sets) into 14 candidate genes (Fig. 2F).

**Fig. 2:**
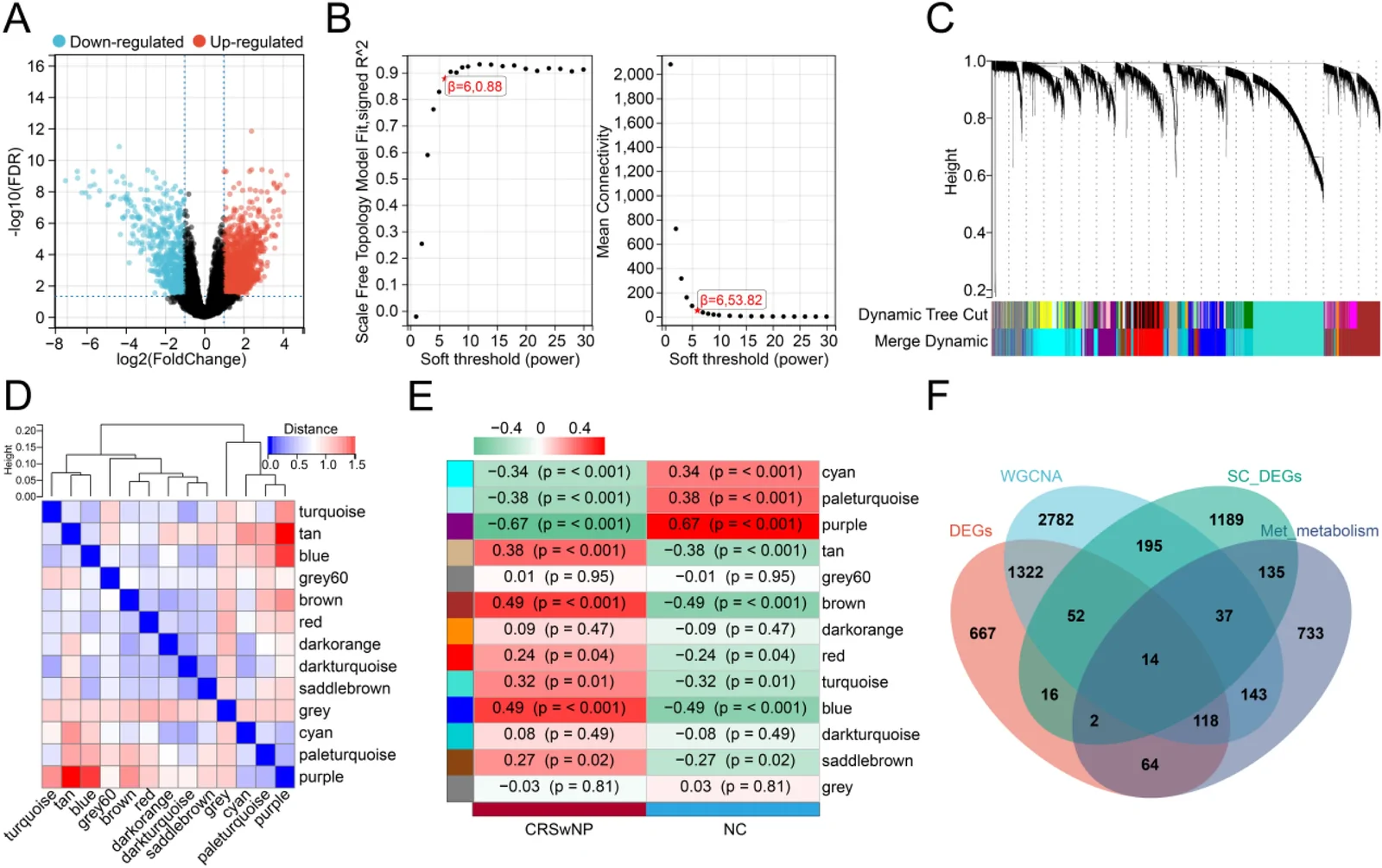
Weighted gene co-expression network analysis. (A) DEG volcano plot; (B) Soft threshold selection; (C) Gene clustering tree; (D) Module correlation heatmap; (E) Module-disease correlation; (F) Venn diagram of candidate genes.

Functional enrichment analysis of the 14 candidate genes demonstrated significant GO enrichment in endopeptidase inhibitor activity and secretory granule lumen (Supplementary Fig. 1A‒C), while KEGG analysis highlighted amino acid biosynthesis, carbon metabolism, and cobalamin transport pathways (Supplementary Fig. 1D) ‒ all consistent with methionine cycle involvement. PPI network analysis identified 42 interactions among 13 candidate genes (Supplementary Fig. 1E), suggesting coordinated co-regulation within a functional module.

### Machine learning screening and validation of key genes

Three independent machine learning algorithms were applied to prioritize the most diagnostically informative genes from the 14 candidates. LASSO identified 7 genes (Fig. 3A), XGBoost ranked 6 genes (Fig. 3B), and SVM-RFE retained 10 genes (Fig. 3C). Intersection of all three algorithms yielded three consensus core genes: CSTB, IGFBP3, and TCN1 (Fig. 3D). SHAP analysis revealed CSTB and IGFBP3 as positive predictors, TCN1 as negative (Fig. 3E‒G). Expression validation showed CSTB and IGFBP3 significantly elevated in CRSwNP, TCN1 reduced (p < 0.01, Fig. 4A‒C). ROC analysis demonstrated excellent diagnostic performance in both cohorts, with AUC values exceeding 0.85 for all three genes (Fig. 4B‒D). With CSTB, IGFBP3, and TCN1 validated as epithelial-enriched biomarkers, subsequent analyses focused on their mechanistic roles within the CRSwNP microenvironment.

**Fig. 3:**
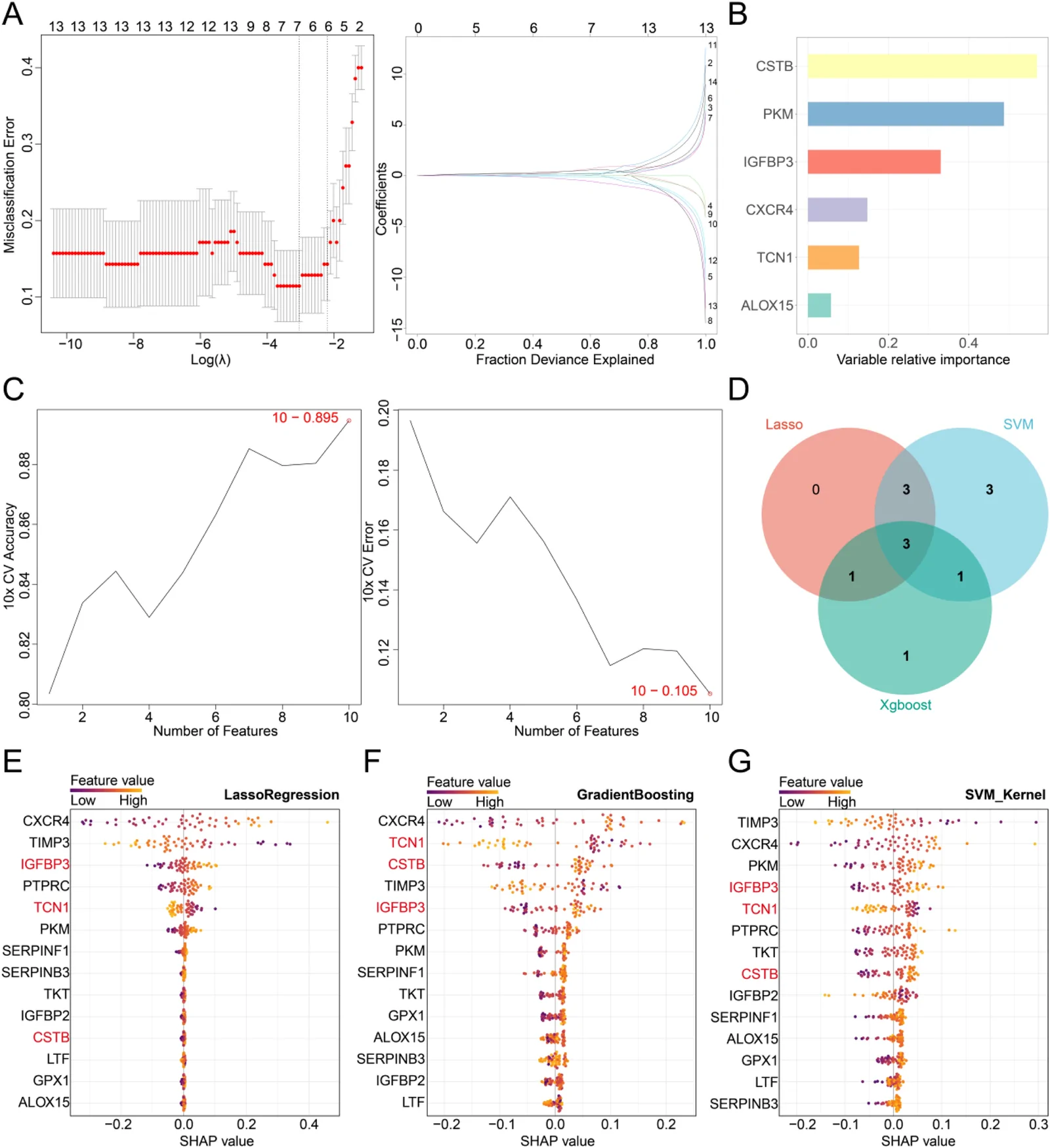
Key target screening based on machine learning algorithms. (A) LASSO results; (B) XGBoost results; (C) SVM-RFE results; (D) Venn diagram; (E–G) SHAP analysis for LASSO, XGBoost, and SVM-RFE models.

**Fig. 4:**
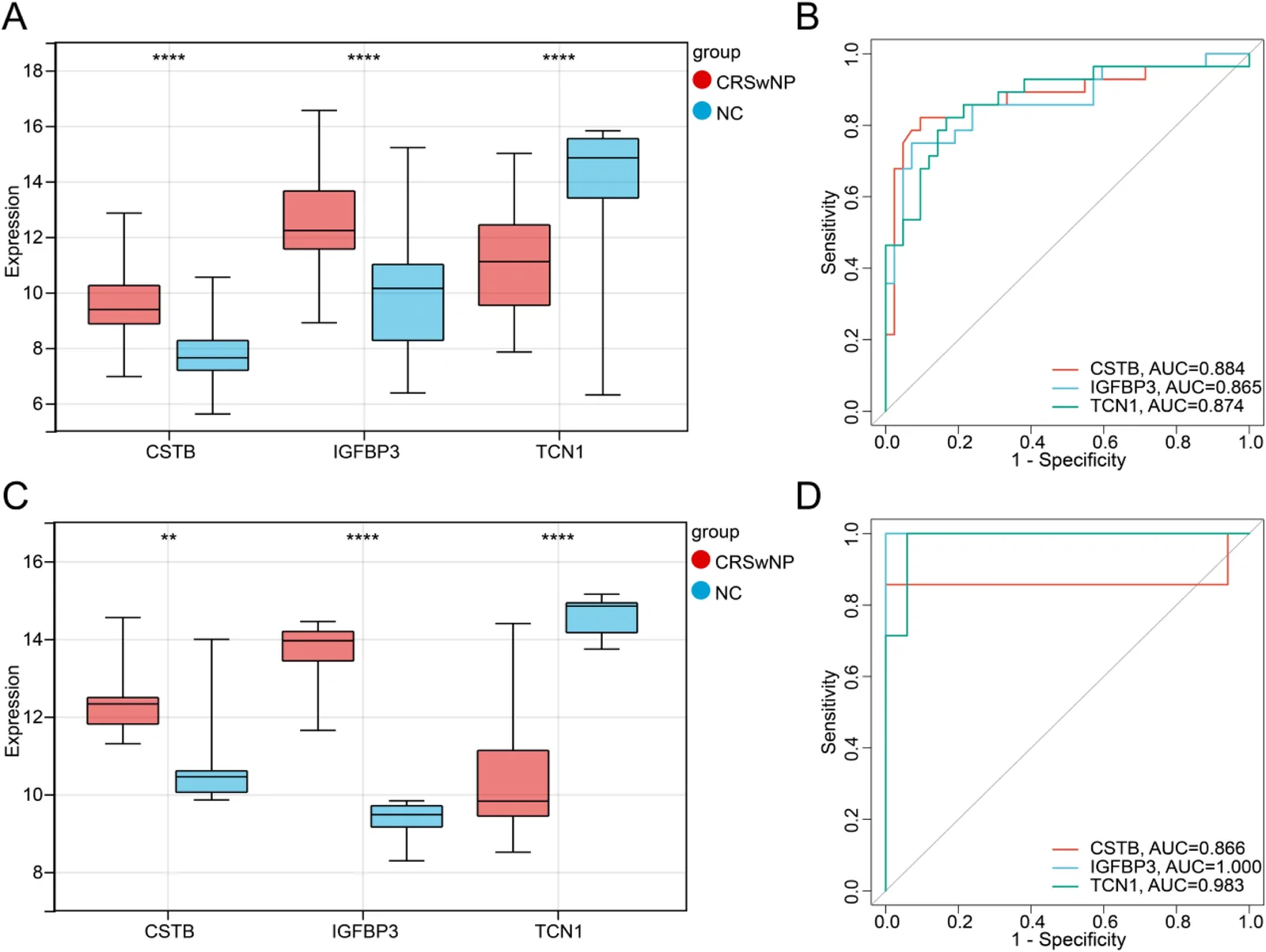
Expression validation and diagnostic performance analysis of key genes. (A) Gene expression in training set; (B) ROC curves in training set; (C) Gene expression in validation set; (D) ROC curves in validation set.

At the single-cell level, all three genes were predominantly expressed in epithelial cells (Figs. 5A, 5 C, 5 E). Within this cell type, CSTB and IGFBP3 were notably upregulated in CRSwNP, while TCN1 showed higher expression in control epithelial cells (Figs. 5B, 5 D, 5 F). Collectively, these converging lines of evidence from bulk transcriptomics, machine learning, and single-cell analysis establish CSTB, IGFBP3, and TCN1 as epithelial cell-enriched, diagnostically reliable biomarkers of CRSwNP.

**Fig. 5:**
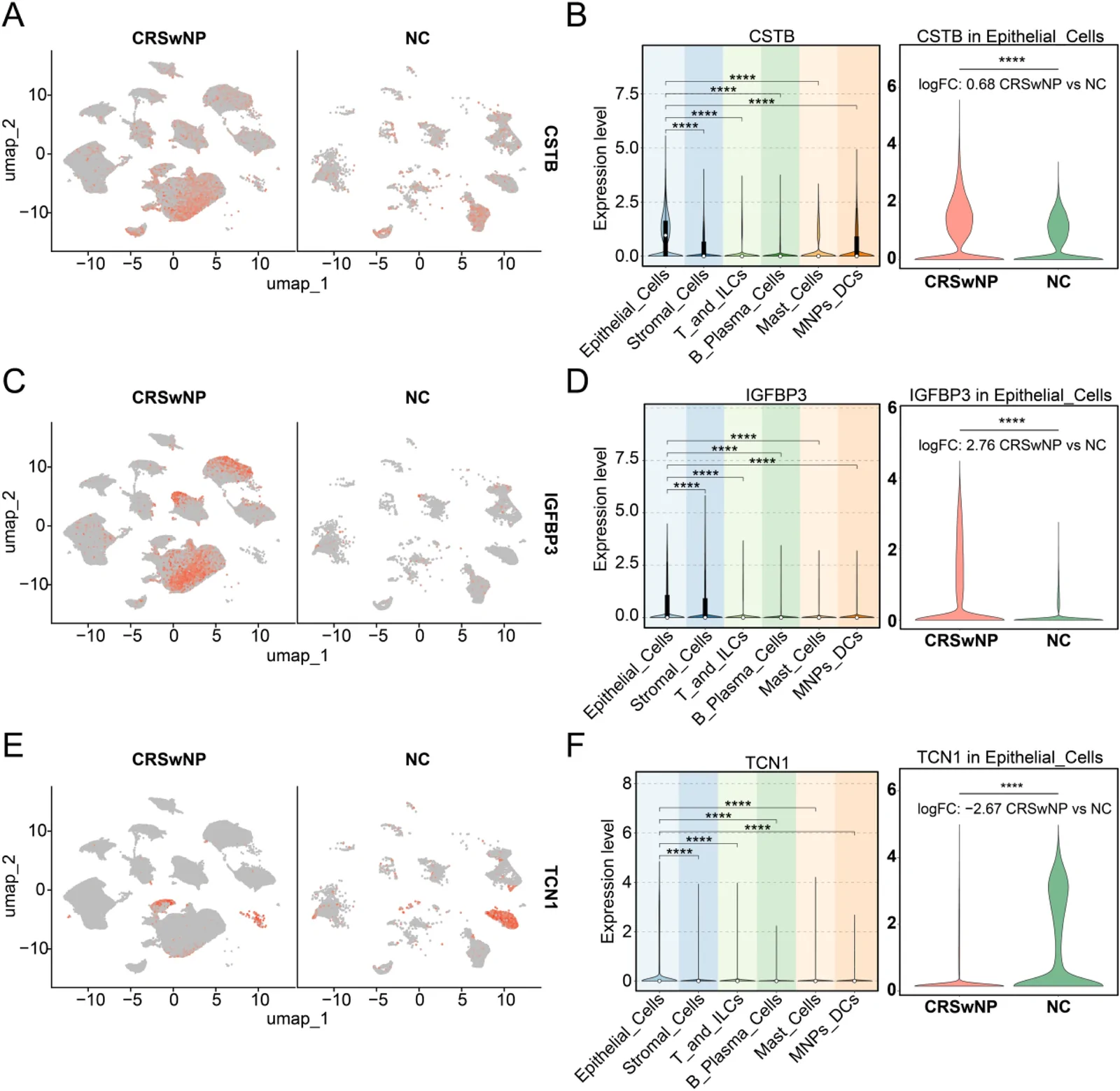
Single-cell level expression analysis of key genes. (A, C, E) UMAP clustering plots showing expression distribution of key genes in single-cell samples: color intensity represents gene expression level, red indicates high expression regions; (B, D, F) Left plots: violin plots showing expression differences of key genes among different cell types; Right plots: violin plots showing expression differences of key genes in epithelial cells between groups.

### Epithelial cell heterogeneity and key gene expression dynamics

CellChat analysis of intercellular communication within the CRSwNP microenvironment revealed that epithelial cells and stromal cells engaged in the greatest number of pairwise interactions (Fig. 6A‒B), with epithelial cells showing strongest communication activity (Fig. 6C‒D). Additionally, MK, EGF, and MIF were the most significant pathways between epithelial cells (Fig. 6E‒H).

**Fig. 6:**
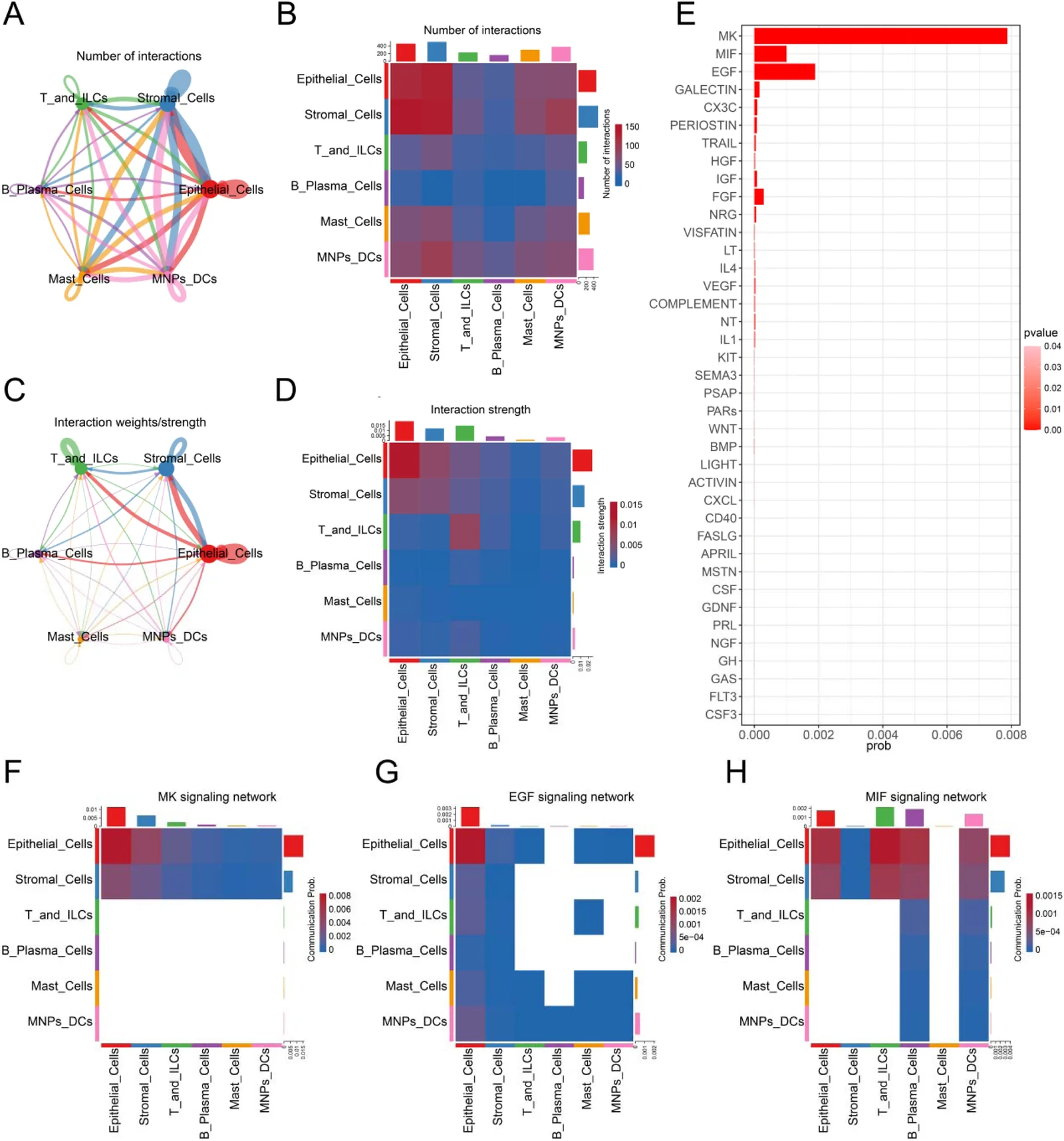
Cell–cell interaction network analysis. (A) Interaction network; (B) Interaction quantity heatmap; (C) Weighted interaction network; (D) Weighted strength heatmap; (E) Key epithelial signaling pathways; (F–H) MK, EGF, and MIF signaling.

After identifying highly variable genes (Supplementary Fig. 2A) and selecting 20 PCs (Supplementary Fig. 2B), SNN clustering identified 10 clusters (Supplementary Fig. 2C‒D). Seven epithelial subtypes were annotated based on marker genes (Supplementary Fig. 2E‒F): basal, club, glandular, goblet, ciliated cells, myoepithelium, and ionocytes. Pseudotime analysis revealed multi-branch differentiation (Supplementary Fig. 2G‒I). Next, we explored the expression changes of key genes during the process of cell differentiation, and found that CSTB and IGFBP3 showed high expression in early differentiation stages (Fig. 7A‒B), while TCN1 was transiently activated mid-differentiation (Fig. 7C), consistent with a role in supporting metabolic homeostasis during a specific phase of epithelial maturation.

**Fig. 7:**
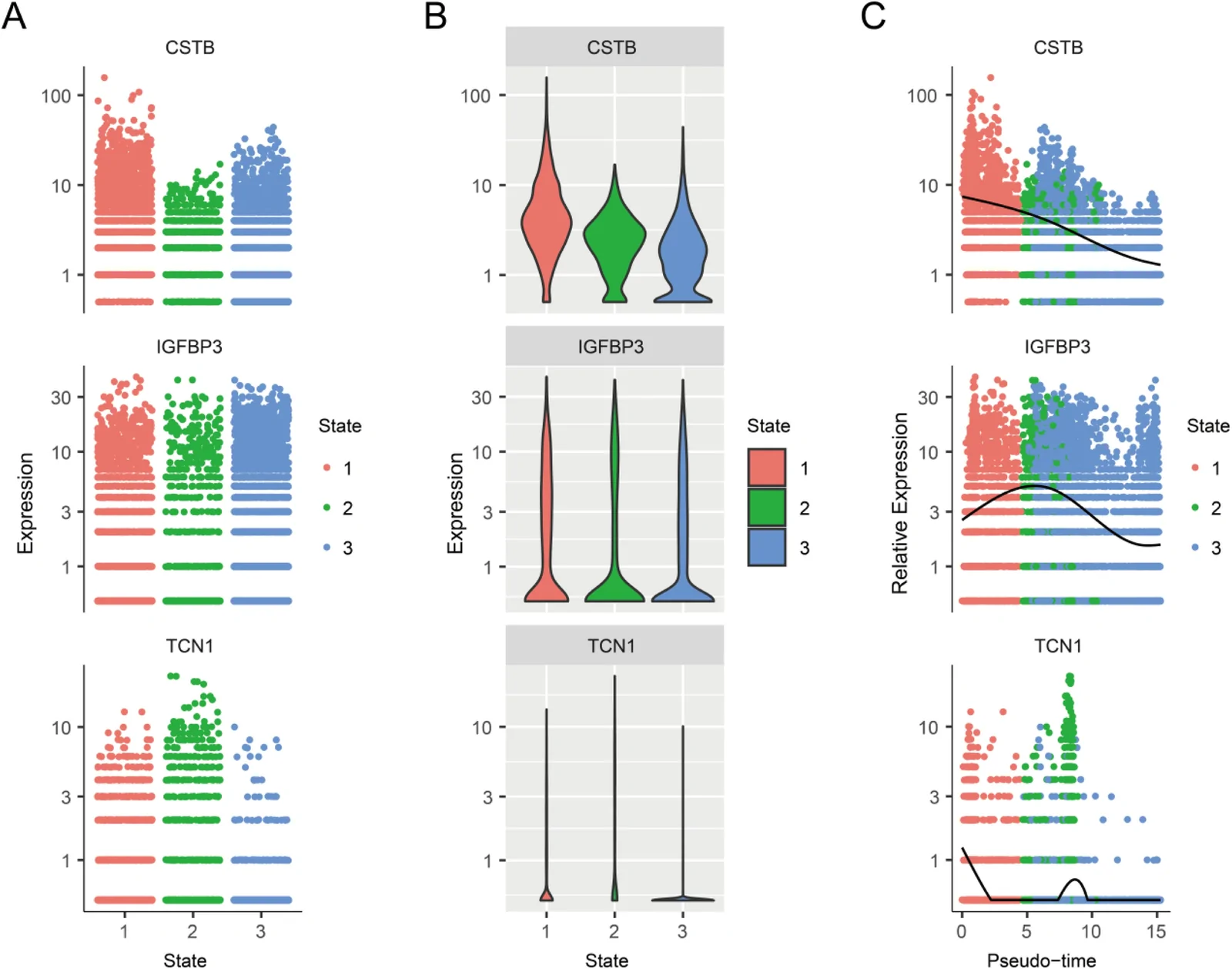
Changes of key genes in epithelial cells in CRSwNP tissues along pseudotime order. (A–C) Expression changes of CSTB, IGFBP3, and TCN1 along pseudotime trajectory.

### Regulatory network and immune microenvironment analysis

To delineate upstream regulatory mechanisms and immune context, we performed single-gene GSEA, ceRNA/TF network construction, and CIBERSORT-based immune deconvolution. Single-gene GSEA demonstrated that all three core genes were enriched in primary immunodeficiency pathways, with CSTB and IGFBP3 showing positive correlation (Supplementary Fig. 3A‒C). The ceRNA network constructed for the three key genes contained 127 nodes and 1228 interactions, with KCNQ1OT1, NEAT1, XIST as hub lncRNAs, and hsa-let-7a/b/d-5p as hub miRNAs (Supplementary Fig. 4A). Upstream transcription factor analysis further identified TFAP2A, SP1, and CTCF as key regulators of core gene expression (Supplementary Fig. 4B), providing candidate nodes for future mechanistic investigation.

CIBERSORT-based deconvolution identified 22 immune subpopulations across CRSwNP and control samples (Supplementary Fig. 5A‒B). CRSwNP showed decreased resting memory CD4 T-cells and plasma cells, but increased M2 macrophages, activated dendritic cells, and neutrophils (p < 0.05, Supplementary Fig. 5C). Correlation analysis revealed that CSTB and IGFBP3 were positively correlated with M2 macrophages and activated dendritic cells but negatively correlated with plasma cells. TCN1 displayed the opposite pattern, positively correlating with plasma cells and resting memory CD4+ T-cells while negatively correlating with innate immune populations (Supplementary Fig. 5D).

### Nomogram construction, drug prediction and molecular docking

A diagnostic nomogram integrating CSTB, IGFBP3, and TCN1 was constructed. The diagnostic nomogram achieved AUC = 0.914, demonstrating strong discriminative performance (Supplementary Fig. 6A‒C).

Candidate therapeutic compounds were predicted via Enrichr (DSigDB) and CMAP databases, yielding five drugs of interest: ambroxol, arbutin, carbamazepine, curcumin, and quercetin. All five exhibited favorable binding free energies (<-5 kcal/moL) against the three core protein targets (Supplementary Table 1). Molecular docking identified the most favorable binding configurations as: ambroxol-CSTB (−5.307 kcal/moL; Fig. 8A‒B), carbamazepine-IGFBP3 (−7.136 kcal/moL; Fig. 8C‒D), and arbutin–TCN1 (−6.686 kcal/moL; Fig. 8E‒F). These results provide preliminary structural evidence supporting the feasibility of repurposing these compounds as therapeutic agents targeting methionine metabolism-associated pathways in CRSwNP.

**Fig. 8:**
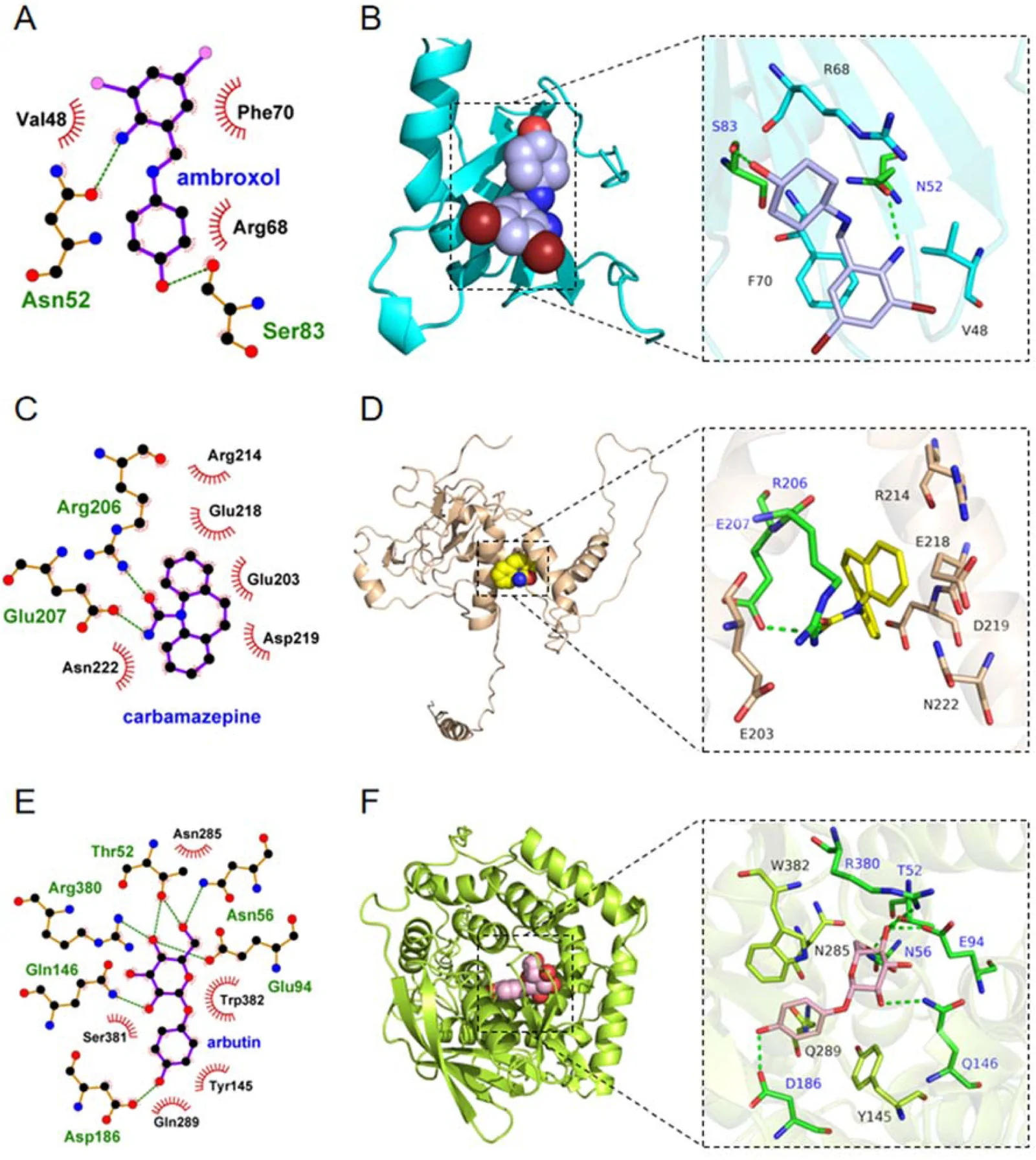
Interaction analysis between small molecule drugs and key targets. (A–B) Ambroxol-CSTB complex in 2D and 3D; (C–D) Carbamazepine-IGFBP3 complex in 2D and 3D; (E–F) Arbutin-TCN1 complex in 2D and 3D.

## Discussion

CRSwNP severely impairs quality of life and healthcare systems.^20^ While methionine metabolism abnormalities contribute to inflammatory diseases,^21^ key genes driving CRSwNP and their mechanisms remain unclear. This study systematically identified CSTB, IGFBP3, and TCN1 as core genes linking methionine metabolism with CRSwNP.

CSTB (cystatin B) encodes a cytoplasmic cysteine protease inhibitor regulating cathepsin activity, with established roles in inflammatory, tumorigenic, and airway disease contexts.^22^, ^23^, ^24^ Elevated cystatin C correlates with Chronic Obstructive Pulmonary Disease (COPD) severity,^25^ while cystatin SN in sputum protects the asthmatic bronchial epithelial barrier by inhibiting sensitizing protease activity and can serve as a potential biomarker for asthma.^26^ More importantly, significant upregulation of cystatin expression has been detected in nasal polyp tissue, and it was found that after its overexpression in the nasal mucosal epithelial layer, it acts as an upstream initiating factor to trigger type 2 inflammatory responses, promoting eosinophil infiltration and cytokine secretion, thereby driving the occurrence and development of CRSwNP.^27^

Consistent with these reported roles, our analyses identified CSTB as significantly overexpressed in CRSwNP epithelial cells, acting as a risk-promoting factor with early differentiation stage expression ‒ findings that collectively implicate CSTB in disease initiation. Mechanistically, aberrant CSTB overexpression excessively suppresses cathepsin activity,^28^ disrupting the protease-antiprotease balance and impairing ECM turnover within the epithelial compartment.^28^^,^^29^ This compromises tight junction integrity and increases epithelial permeability,^30^ facilitating allergen penetration and triggering TSLP, IL-25, and IL-33 alarmin release. These alarmins activate ILC2s and prime type 2 dendritic cells, orchestrating downstream IL-4, IL-5, and IL-13 production, eosinophil recruitment, and IgE-mediated sensitization that defines eosinophilic CRSwNP.^31^ Paradoxically, this “overprotection” impairs efferocytotic clearance of accumulated eosinophils,^32^^,^^33^ and drives fibrosis progression and self-sustaining chronic inflammation rather than facilitating resolution. The positive correlation of CSTB with M2 macrophages and activated dendritic cells supports its central role in maintaining the chronic inflammatory microenvironment, as cathepsin-impaired efferocytosis skews macrophage polarization toward pro-fibrotic M2 phenotypes while CSTB-induced alarmin release continuously activates dendritic cells toward Th2-polarizing programs. Mechanistically, SAM depletion resulting from impaired methionine cycle flux may reduce DNMT-mediated CpG methylation at the CSTB promoter, providing an epigenetic basis for its transcriptional upregulation in CRSwNP epithelium. Notably, this is the first study to identify CSTB as a differentially expressed and functionally significant gene specifically in CRSwNP tissue, linking it to methionine metabolism dysregulation in this disease context.

Insulin-like Growth Factor Binding Protein 3 (IGFBP3) regulates IGF bioavailability and exerts IGF-independent biological functions encompassing cell proliferation, apoptosis, and inflammatory responses.^34^ IGFBP3 upregulation has been reported in asthmatic airway epithelial cells,^35^ and its expression is modulated by the circMETTL9/EIF4A3 axis in inflammatory and oxidative stress contexts.^36^ In idiopathic pulmonary fibrosis, IGFBP3 drives inflammation and Epithelial-Mesenchymal Transition (EMT) through the p53/IGFBP3 pathway, with its expression level correlating with fibrotic progression.^37^ Our study confirms IGFBP3 upregulation in CRSwNP, consistent with its pro-fibrotic and immune-modulatory roles described in other chronic inflammatory airway diseases. In the polyp microenvironment, elevated IGFBP3 sequesters IGF-1, reducing IGF-1R-mediated PI3K/AKT survival signaling in epithelial cells^38^^,^^39^ and compounding the barrier dysfunction driven by CSTB. Simultaneously, IGFBP3 upregulation activates TGF-β1 secretion through the p53/IGFBP3 axis,^40^ engaging SMAD2/3 pathways to promote EMT and driving fibroblast-to-myofibroblast transition with consequent collagen deposition and subepithelial fibrosis characteristic of nasal polyp histology.^41^^,^^42^ Furthermore, TGF-β elevation reinforces M2 macrophage polarization and suppresses cytotoxic immune responses, establishing the immunosuppressive stromal niche that impedes inflammation resolution.^43^^,^^44^ The positive correlation of IGFBP3 with innate immune cells (particularly M2 macrophages and activated dendritic cells) in this study supports this speculation.

TCN1 encodes transcobalamin I, which is responsible for the transport and stabilization of vitamin B12. Vitamin B12 is an important cofactor for methionine synthase, directly participating in the methionine cycle and one-carbon metabolism.^45^ Vitamin B12 deficiency is associated with airway hyperresponsiveness and increased asthma risk,^46^ and decreased vitamin B12 levels are associated with mucosal inflammation severity and impaired immune homeostasis in inflammatory bowel disease.^47^ TCN1 downregulation in CRSwNP, confirmed in our analysis as a protective factor, has direct mechanistic implications for methionine cycle integrity. Reduced TCN1 expression decreases intracellular methionine bioavailability,^48^ impairs MTR activity, and leads to dual pathogenic consequences. Homocysteine accumulates and activates NF-κB-mediated pro-inflammatory signaling and mitochondrial ROS production.^49^^,^^50^ This epigenetic dysregulation broadly deregulates type 2 immune polarization programs and maintains the CRSwNP inflammatory phenotype through persistent epigenetic mechanisms.^51^ TCN1 positively correlates with plasma cells and resting memory CD4^+^ T-cells, and negatively correlates with innate immune cells, reflecting the methionine dependence of adaptive immune function. Therefore, TCN1 downregulation selectively impairs adaptive immunity and pathogen-specific recall ability, explaining the chronic relapsing characteristics of CRSwNP. These three genes converge on a unified methionine-immune axis: TCN1 downregulation impairs SAM production and promotes homocysteine accumulation, epigenetically derepressing CSTB and IGFBP3, which collectively disrupt epithelial barrier integrity and drive type 2 immune polarization in CRSwNP.

Seven epithelial subtypes were identified, with goblet and ciliated cell abnormalities contributing to mucus hypersecretion and impaired clearance,^52^^,^^53^ and basal cell activation driving tissue remodeling.^54^ The high expression of CSTB at early differentiation stages suggests involvement in epithelial fate determination ‒ potentially skewing differentiation toward inflammatory subtypes through protease-mediated microenvironmental modification. Complementing this cellular heterogeneity analysis, cell communication analysis revealed that epithelial cells exhibit the highest intercellular communication activity in CRSwNP, with MK, EGF, and MIF pathways as dominant mediators of cell migration, proliferation, and inflammatory amplification.^55^ Together, these data reframe epithelial cells as active orchestrators ‒ rather than passive targets ‒ of CRSwNP pathogenesis, operating through a coordinated network of subtype-specific gene expression, fate-determining epigenetic reprogramming, and paracrine inflammatory signaling.

The ceRNA network analysis revealed a complex regulatory network based on key genes. The let-7 family is highly expressed in various systems and participates in the progression of diseases.^56^ Furthermore, it plays important roles in cell cycle, metabolism, and immune regulation.^57^ Transcription factor analysis identified TP53, E2F2, and USF1. E2F2 participates in cell cycle regulation and can enhance tumor cell proliferation, angiogenesis, and invasiveness^58^; the role of USF1 in metabolic gene transcription is directly linked to cell proliferation pathways.^59^ The regulatory relationships between these transcription factors and key genes provide important clues for in-depth understanding of the upstream regulatory mechanisms of methionine metabolism abnormalities.

This study conducted a prediction of targeted drugs for key genes. Ambroxol, beyond its well-known mucolytic activity, has been shown to modulate lysosomal cathepsin activity and suppress NF-κB-mediated inflammatory cascades.^60^ Curcumin exerts broad epigenetic regulatory effects, which directly align with the TCN1-mediated epigenetic deregulation identified in this study.^61^ Arbutin exhibits antioxidant and anti-inflammatory properties that may counteract NF-κB activation.^62^ Quercetin, a polyphenolic flavonoid, modulates both IGF signaling and type 2 cytokine production.^63^ However, the precise mechanisms by which these compounds exert therapeutic effects on CRSwNP through modulation of CSTB, IGFBP3, and TCN1 warrant further experimental validation.

Limitations include: 1) Lack of in vitro validation of methionine restriction, SAM deficiency, or B12 deficiency effects on key gene expression; 2) Absence of animal models validating causal roles; 3) Cross-sectional design limiting temporal relationship determination; 4) Lack of prospective validation of diagnostic model utility; 5) Molecular docking excluding pharmacokinetics and therapeutic effect considerations. Future research should validate key gene functions through genetic manipulation in CRSwNP animal models, explore molecular mechanisms regulating epithelial barriers and immune responses, conduct drug repositioning studies of candidate compounds (ambroxol, curcumin) with in vitro/*vivo* efficacy evaluation, and integrate multi-omics data to construct methionine metabolism-related precision classification systems for CRSwNP.

## Funding

The author received no financial support for the research, authorship, and/or publication of this article.

## Data availability

The authors declare that all data are available in repository.

## Declaration of competing interest

The authors declare no conflicts of interest.
