# Supplementary material for: Exploring the role and mechanism of methionine metabolism in chronic rhinosinusitis with nasal polyps based on bioinformatics analysis
Source: Braz J Otorhinolaryngol. 2026 Oct 31;93(1):101917. doi: 10.1016/j.bjorl.2026.101917 (PMC13743948; doi:10.1016/j.bjorl.2026.101917)
Supplement: Supplementary file 1 [file mmc1.docx]

BJORL-D-25-00447_Supplementary Material

**Supplementary Figure 1. Functional Enrichment Analysis**

(A-C) Bar charts of BP (A), CC (B), MF (C) GO terms enriched by candidate genes (top 10 most significant terms); (D) Bar chart of KEGG pathways enriched by candidate genes; (E) PPI network analysis of candidate genes.

**Supplementary Figure 2. Heterogeneity Analysis of Epithelial Cells in CRSwNP Tissues**

(A) Highly Variable Genes (HVGs) screening results: scatter plot showing relationship between average gene expression levels and normalized variance, red dots represent screened highly variable genes (top 2000), black dots represent non-variable genes; (B) Principal component analysis (PCA) variance explanation plot: standard deviation distribution showing contributions of each principal component (PCs) to data variance; (C) Dimensionality reduction results based on principal component analysis (PCA), showing distribution of different cell clusters in low-dimensional space; (D) Shared nearest neighbor (SNN) algorithm clustering results, showing UMAP distribution of different cell clusters in all samples; (E) Expression heatmap of cell type marker genes: horizontal axis represents feature genes, vertical axis represents annotated major cell types, bubble size indicates proportion of cells expressing genes, color intensity represents average gene expression level; (F) Cell annotation of 10 cell subpopulations combined with known cell type marker genes; (G) Dispersion analysis of epithelial cell gene expression, red line shows negative correlation trend, black dots represent screened high dispersion genes; (H) Cell differentiation trajectory analysis, numbers represent differentiation branch points; (I) Density distribution plots of different epithelial cell subtypes along pseudotime order.

**Supplementary Figure 3. Single-Gene GSEA Analysis Based on Training Set**

(A) Single-gene GSEA analysis of CSTB based on training set; (B) Single-gene GSEA analysis of IGFBP3 based on training set; (C) Single-gene GSEA analysis of TCN1 based on training set.

**Supplementary Figure 4. ceRNA Regulatory Network and Transcription Factor Analysis**

(A) ceRNA regulatory network related to key genes CSTB, IGFBP3, and TCN1: red nodes represent mRNA, yellow nodes represent miRNA, green nodes represent lncRNA; (B) Transcription factor regulatory network of key genes CSTB, IGFBP3, and TCN1: red nodes represent mRNA, light green nodes represent predicted transcription factors (TF).

**Supplementary Figure 5. Immune Infiltration Analysis**

(A) Infiltration proportions of immune cells among samples; (B) Correlation analysis heatmap between immune cells, *P < 0.05, **P < 0.01, ***P < 0.001; (C) Difference analysis of immune cell infiltration proportions between CRSwNP and NC group samples *P < 0.05, **P < 0.01, ***P < 0.001; (D) Correlation heatmap between key genes and immune cells, *P < 0.05, **P < 0.01.

**Supplementary Figure 6. Nomogram Based on Key Genes**

(A) Nomogram model based on key genes CSTB, IGFBP3, TCN1; (B) ROC curve of predictive model; (C) Decision curve analysis of the model.

**Supplementary Table 1. Binding Free Energies Between Key Genes and Small Molecule Drugs.**

| **Compound** | **Target** | **Binding free energies (kcal/mol)** |
| --- | --- | --- |
| ambroxol | CSTB | -5.307 |
| carbamazepine | IGFBP3 | -7.136 |
| curcumin | IGFBP3 | -6.864 |
| quercetin | IGFBP3 | -6.986 |
| arbutin | TCN1 | -6.686 |
